# Identification of a ferritin-like protein of *Listeria monocytogenes* as a mediator of β-lactam tolerance and innate resistance to cephalosporins

**DOI:** 10.1186/1471-2180-12-278

**Published:** 2012-11-24

**Authors:** Agata Krawczyk-Balska, Julia Marchlewicz, Dorota Dudek, Katarzyna Wasiak, Anna Samluk

**Affiliations:** 1 Department of Applied Microbiology, Faculty of Biology, University of Warsaw, Miecznikowa 1, 02-096, Warsaw, Poland

**Keywords:** *Listeria monocytogenes*, Tolerance to β-lactams, Resistance to cephalosporins

## Abstract

**Background:**

The food-borne pathogen *Listeria monocytogenes* is the causative agent of listeriosis. The β-lactam antibiotics penicillin G and ampicillin are the current drugs of choice for the treatment of listerial infections. While isolates of *L*. *monocytogenes* are susceptible to these antibiotics, their action is only bacteriostatic and consequently, this bacterium is regarded as tolerant to β-lactams. In addition, *L*. *monocytogenes* has a high level of innate resistance to the cephalosporin family of β-lactams frequently used to treat sepsis of unknown etiology. Given the high mortality rate of listeriosis despite rational antibiotic therapy, it is important to identify genes that play a role in the susceptibility and tolerance of *L*. *monocytogenes* to β-lactams.

**Results:**

The *hly*-based promoter trap system was applied to identify penicillin G-inducible genes of *L*. *monocytogenes*. The results of reporter system studies, verified by transcriptional analysis, identified ten penicillin G-inducible genes. The contribution of three of these genes, encoding a ferritin-like protein (*fri*), a two-component phosphate-response regulator (*phoP*) and an AraC/XylS family transcriptional regulator (*axyR*), to the susceptibility and tolerance of *L*. *monocytogenes* to β-lactams was examined by analysis of nonpolar deletion mutants. The absence of PhoP or AxyR resulted in more rapid growth of the strains in the presence of sublethal concentration of β-lactams, but had no effect on the MIC values or the ability to survive a lethal dose of these antibiotics. However, the Δ*fri* strain showed impaired growth in the presence of sublethal concentrations of penicillin G and ampicillin and a significantly reduced ability to survive lethal concentrations of these β-lactams. A lack of Fri also caused a 2-fold increase in the sensitivity of *L*. *monocytogenes* to cefalotin and cephradine.

**Conclusions:**

The present study has identified Fri as an important mediator of β-lactam tolerance and innate resistance to cephalosporins in *L*. *monocytogenes*. PhoP and AxyR are probably involved in transmitting signals to adjust the rate of growth of *L*. *monocytogenes* under β-lactam pressure, but these regulators do not play a significant role in susceptibility and tolerance to this class of antibiotics.

## Background

*Listeria monocytogenes* is a food-borne facultative intracellular pathogen that causes a wide spectrum of clinical disease in humans, ranging from mild influenza-like illness and gastroenteritis to severe listeriosis with meningitis, which is frequently accompanied by septicemia and meningoencephalitis. While listeriosis may occur in otherwise healthy individuals, those primarily at risk are immunocompromised patients, pregnant women, the very young and the elderly [1]. The antibiotics of choice in the treatment of listeriosis are the β-lactams penicillin G and ampicillin, alone or in combination with gentamicin. However, despite the use of antibiotic therapy, up to one-third of patients die [2].

In general, isolates of *L*. *monocytogenes* are susceptible to β-lactam antibiotics, except for members of the cephalosporin family. However, for most isolates, there is a large gap between the MIC (minimal inhibitory concentration) and MBC (minimal bactericidal concentration) values of β-lactam antibiotics. Consequently, *L*. *monocytogenes* is regarded as tolerant to all β-lactams [2,3]. Furthermore, the high level of innate resistance of *L*. *monocytogenes* to cephalosporins may be especially significant since members of this family of β-lactams are frequently used to treat sepsis of unknown etiology.

Tolerance to β-lactams and innate resistance to cephalosporins are among the most important factors contributing to the not infrequent ineffectiveness of antibiotic therapy of listeriosis. In an effort to decrease the significant human and economic costs associated with listeriosis, the development of methodologies to reduce the survival and growth of *L*. *monocytogenes* during infection is the focus of much research effort. One of the primary goals is to characterize the mechanisms of susceptibility and tolerance of *L*. *monocytogenes* to β-lactams.

To date, a number of genes that play a role in the innate resistance of *L*. *monocytogenes* to cephalosporins have been identified. Of these, *lmo0441*, *lmo2229* and *lmo2754* encode penicillin binding proteins that are the classical target enzymes for β-lactam antibiotics [4]. Other examples of genes contributing to innate resistance are *mdrL*, which encodes an antibiotic efflux pump [5], *telA* a gene homologous to tellurite resistance loci [6], *anrAB*, which encodes a putative multidrug resistance transporter [7] and *lmo1416* a homolog of *Enterococcus faecium vanZ*[8]. In addition, the two-component systems (TCSs) CesRK and LisRK have been identified as key mediators involved in the innate resistance of *L*. *monocytogenes* to cephalosporins [9,10]. Most recently, genome-wide transcriptional studies have confirmed the crucial role of LisRK and CesRK in the response of *L*. *monocytogenes* to β-lactams and have demonstrated that two other TCSs, LiaSR and VirRS, are also linked to this response [11]. The mechanisms of tolerance of *L*. *monocytogenes* to cell envelope-acting antimicrobial agents are much more poorly characterized than the mechanisms of innate resistance to cephalosporins. To date, only the alternative sigma factor SigB has been shown to determine the tolerance of *L*. *monocytogenes* to β-lactams [12].

It seems reasonable to assume that certain genes that are important for the survival and growth of bacteria in the presence of cell envelope-acting antibiotics are induced during treatment with these antimicrobial agents. Several studies have provided evidence to support this assumption in the case of *L*. *monocytogenes*. Stack et al. [13] showed that *htr*A, encoding an HtrA-like serine protease, is essential for the growth of *L*. *monocytogenes* in the presence of penicillin G, and that this gene is more efficiently transcribed when this β-lactam is present. Gottschalk et al. [8] demonstrated that the transcription of several cell wall-related genes (controlled by the CesRK two-component system) is induced by β-lactam and glycopeptide antibiotics. Three of these genes, *lmo1416*, *lmo2210* and *lmo2812*, play a significant role in the survival of the bacterium in the presence of cell wall-acting antibiotics. More recently, Nielsen et al. [11] showed the same relationship between the induction of expression and significance of *lmo2442* and *lmo2568* genes in the susceptibility of *L*. *monocytogenes* to the β-lactam antibiotic cefuroxime. These observations prompted us to attempt to identify *L*. *monocytogenes* genes induced in the presence of penicillin G, in order to learn more about mechanisms of tolerance to this class of antibiotic. For this purpose, a promoter-trap system based on a promoterless plasmid-borne copy of the *hly* gene encoding listeriolysin O (LLO) was employed. This system has been used previously to identify *L*. *monocytogenes* promoters that are either constitutive or specifically induced during in vivo infection [14].

In the course of this study, ten penicillin-G inducible genes were identified. The upregulated expression of these genes under penicillin G pressure was verified by transcriptional analysis. Three of the identified genes, namely *fri*, *phoP* and *axyR*, were selected for further investigation. The *fri* gene encodes a non-heme, iron-binding ferritin-like protein (Fri) that belongs to the Dps (*D*NA-binding *p*roteins from *s*tarved cells) family of proteins, which play important roles in the response to multiple stresses in many bacterial species (reviewed recently in [15]). Gene *phoP* encodes a two-component phosphate-response regulator homologous to *B*. *subtilis phoP*, which plays a crucial role in controlling the biosynthesis of teichoic acid, a key component of the gram-positive bacterial cell wall [16]. Gene *axyR* encodes a transcriptional regulator from the AraC/XylS family, some members of which are involved in tolerance to antibiotics [17]. The susceptibility and tolerance to β-lactams of nonpolar deletion mutants in the three selected genes was examined. It was revealed that Fri is a mediator of tolerance to penicillin G and ampicillin, as well as of resistance to some cephalosporins, including cefalotin and cephradine. The identification of a locus that contributes to tolerance to β-lactams used in the treatment of listeriosis and that is relevant to the innate resistance of *L*. *monocytogenes* to cephalosporins is notable in light of the clinical use of these antibiotics.

## Results

### Screening of *L*. *monocytogenes* genomic libraries for penicillin G-inducible promoters

Genomic DNA of *L*. *monocytogenes* was fragmented using four different procedures and the obtained chromosomal fragments were cloned upstream of the promoterless *hly* gene in vector pAT28-*hly*. This vector has previously been used to identify constitutive as well as inducible promoters of *L*. *monocytogenes*[14]. It was chosen for the identification of penicillin G-inducible promoters because the plasmid is present in *L*. *monocytogenes* at high copy number, which permits the selection of even relatively weak promoters driving *hly* expression. Penicillin G was selected for this study because it is widely used as the antibiotic of choice for the treatment of listerial infections [2]. The four genomic libraries were introduced into *L*. *monocytogenes* EGDΔ*hly* by electroporation and transformed strains in which putative promoters were trapped upstream of *hly*, were identified by the creation of hemolytic zones on blood agar plates. To determine whether expression was induced by penicillin G, the strains were replica plated on blood agar plates with or without this antibiotic. Penicillin G was used at a concentration (0.03 μg/ml) that permitted the growth of *L*. *monocytogenes* EGD even under prolonged incubation, but which exerted a deleterious effect on the bacteria, as evidenced by a reduced growth rate and lower cell number compared with cultures without the antibiotic. Strains producing larger hemolytic zones on blood agar plates supplemented with penicillin G were identified. Inducible expression of the promoter-*hly* fusions in the selected strains in response to the addition of penicillin G was further quantified using a hemolytic activity assay. In the presence of penicillin G a significant increase in hemolytic activity produced by nine of the selected strains was observed (Table 1).

**Table 1:** **Expression of promoter-*hly *fusions in response to the addition of penicillin G as determined by a hemolytic activity assay**

**Hemolytic activity**^***a***^									
**Strain**	**15**^***b***^	**18**^***b***^	**37**^***c***^	**41**^***b***^	**195**^***d***^	**198**^***c***^	**199**^***c***^	**201**^***c***^	**203**^***d***^
**K**	10.2 ± 2.6	8.7 ± 1.6	13.2 ± 3.8	20.7 ± 2.5	30.8 ± 1.2	20.3 ± 1.4	12.2 ± 0.6	21.5 ± 1.3	19.6 ± 1.1
**PenG**	20.4 ± 1.9^**^	13.3 ± 0.3^*^	32.5 ± 4.5^**^	36.1 ± 1.9^**^	54.8 ± 1.8 ^**^	29.5 ± 1.7^*^	33.9 ± 1.6^**^	28.5 ± 1.7^**^	55.5 ± 3.4^**^

^*a*^ The hemolytic activity was measured for selected strains carrying promoter-*hly* fusions grown for 2 h in the presence (PenG) or absence (K) of penicillin G (0.03 μg/ml), using ^*b*^0.5%, ^*c*^1% or ^*d*^2% suspensions of SRBC. The results are the average of three independent experiments, each performed in triplicate ± the standard deviation. Asterisks indicate significant differences according to Student’s *t* test (^*^, *P* < 0.05; ^**^, *P* < 0.01).

### Analysis of trapped chromosomal DNA fragments in strains showing penicillin G-inducible *hly* expression

**Figure 1:**
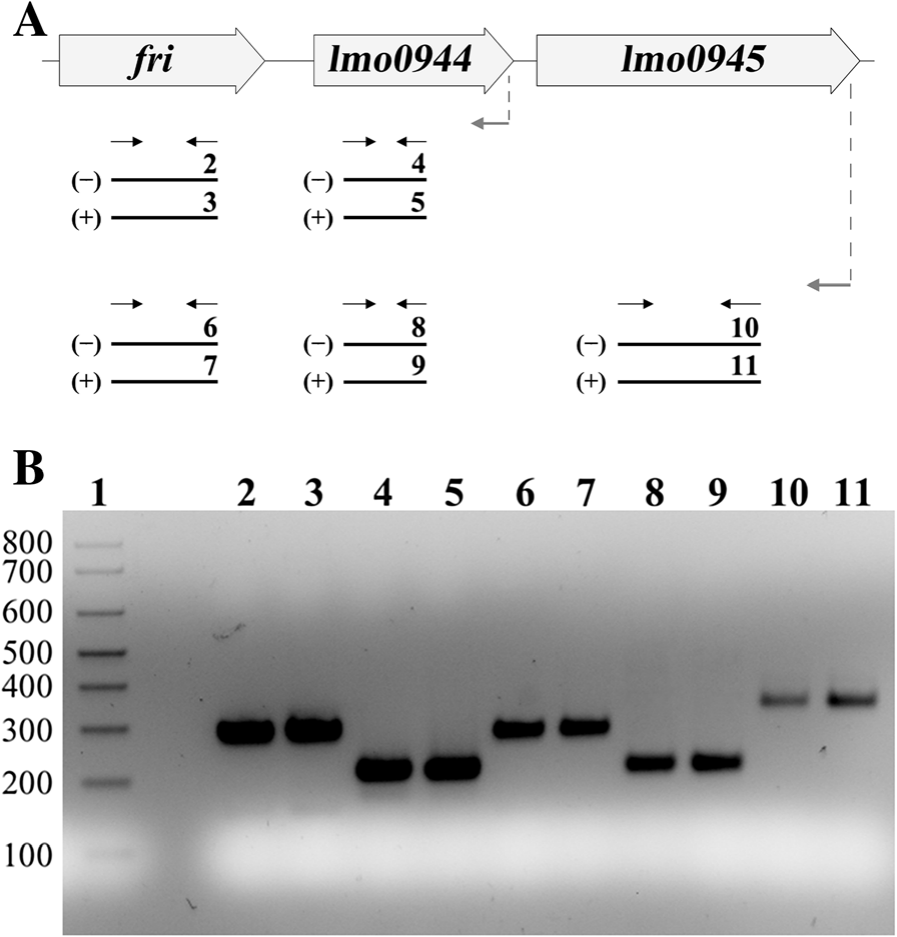
**Analysis of cotranscription of *fri, lmo0944 *and *lmo0945 *genes by RT-PCR.** (**A**) Scheme for transcriptional analysis of the genomic region comprising the *fri*, *lmo0944* and *lmo0945* genes. The template RNA was isolated from exponential-phase cultures of *L*. *monocytogenes* EGD grown in BHI broth at 37°C without antibiotics or with 0.09 μg/ml penicillin G. Gray arrows indicate the positions of the primers used in RT reactions and black arrows indicate the positions of primers used for PCR. Black lines labeled 2 through 11 show the positions of the expected products. The RT-PCR product labels correspond to the numbering of the agarose gel lanes in panel **B**. (-) or (+) indicate the expected products amplified using the RNA templates isolated from cells grown without antibiotics or with penicillin G, respectively. (**B**) The products obtained in RT-PCR reactions. The expected size of the amplified fragments of *fri*, *lmo0944* and *lmo0945* was 288 bp, 212 bp and 332 bp, respectively. A 100-bp ladder (lane 1) is shown as a size marker. In all cases, control PCRs were performed to confirm the complete removal of DNA from the RNA preparations prior to reverse transcription (data not shown).

The chromosomal fragments carrying penicillin G-inducible promoters were sequenced and compared with the *L*. *monocytogenes* EGD-e genome. In the case of seven strains, namely 15, 18, 37, 198, 199, 201 and 203 (Table 2), this analysis identified single genes as the source of the trapped chromosomal DNA fragments. In the case of strain 195, the trapped fragment was comprised of sequences originating from two genes, *lmo2095* and *lmo2096*, both present in the opposite transcriptional orientation to the reporter gene. It was reasoned that the identified promoter might originate from a divergently transcribed gene positioned immediately upstream of the cloned fragment, but examination of the genome sequence showed that the two preceding genes, *lmo2097* and *lmo2098*, are in the same orientation as *lmo2095* and *lmo2096*. Thus, the identified promoter could not direct the expression of any of these genes and for this reason it was excluded from further investigations. In the case of strain 41, the trapped chromosomal fragment contained the full sequence of genes *lmo0943* (*fri*) and *lmo0944* plus sequences upstream of these genes, as well as a fragment of the sequence preceding gene *lmo0945*, which is in the same transcriptional orientation. Thus, on the basis of simple sequence analysis it was not possible to identify which promoter was directing *hly* expression in this strain. In an attempt to clarify this situation, the possible cotranscription of *fri*, *lmo0944* and *lmo0945* was examined by RT-PCR. The three anticipated PCR products were amplified from cDNA generated by reverse transcription using primers specific for genes *lmo0945* and *lmo0944*, which demonstrated that *fri*, *lmo0944* and *lmo0945* are cotranscribed in both non-stressed cells and in cells grown under penicillin G pressure (Figure 1). Consequently, each of these genes was analyzed further.

**Table 2:** **Description of *L.*. *monocytogenes *chromosomal DNA fragments trapped upstream of the *hly *gene in strains exhibiting penicillin-G induced hemolysis**

**Strain**	**Cloned fragment**	
	**Localization**^***a***^**[5**^**′**^** → 3**^**′**^**]**	**Comments**^***b***^
15	2018205-2016470	1347-bp fragment of *lmo1942*, intergenic region (320 bp), **68-bp fragment of *lmo1941***
18	2907708-2906509	556-bp fragment of *lmo2821*, intergenic region (131 bp), **432-bp fragment of *axyR***
37	1712064-1710832	392-bp fragment of *lmo1661*, intergenic region (404 bp), **436-bp fragment of *leuS***
41	978762-980082	93-bp fragment of *lmo0942*, intergenic region (202 bp), gene *fri* (471 bp), intergenic region (233 bp), gene *lmo0944* (303 bp), **18-bp intergenic region preceding *lmo0945***
195	2174937-2175826	484-bp fragment of *lmo2095*, intergenic region (20 bp), **386-bp fragment of *lmo2096***
198	1664108-1664654	362-bp fragment of *lmo1621*, intergenic region (106 bp), **79-bp fragment of *lmo1622***
199	2577843-2576921	147-bp fragment of *lmo2502*, intergenic region (151 bp), **625-bp fragment of *phoP***
201	1234555-1233755	89-bp fragment of *lmo1213*, intergenic region (31 bp), gene *lmo1212* (531 bp), intergenic region (89 bp), **60-bp fragment of *lmo1211***
203	1093048-1092427	48-bp fragment of *lmo1066*, intergenic region (131 bp), **442-bp fragment of *lmo1065***

^*a*^ Nucleotide position in genome of *L*. *monocytogenes* EGD (Acc. No. NC_003210) given in the same orientation as the reporter gene.

^*b*^Genes/fragments of genes and intergenic region present in the trapped fragments, with the sequence located directly upstream of the 5′ end of the *hly* gene marked in bold, while the genes/fragments of genes in the same orientation as this reporter gene are underlined.

The genes whose promoters were identified as responsible for increased *hly* expression in the presence of penicillin G were further characterized (Table 3) and four of them were found to have established functions. Gene *phoP* encodes a transcriptional regulator of the two-component system PhoPR, *fri* encodes a non-heme iron-binding ferritin involved in adaptation to atypical conditions, *leuS* encodes a leucyl-tRNA synthetase engaged in protein synthesis, and *axyR* encodes a putative transcriptional regulator with homology to AraC/XylS regulators. The functions of the proteins encoded by the six other identified penicillin G-inducible genes are unknown, but some predictions could be made on the basis of their homology to proteins with putative functions and/or the presence of domains possessing a specific function. Proteins Lmo1941 and Lmo0945 possess putative signal sequences and thus could represent surface proteins of *L*. *monocytogenes*. Lmo0945 shows homology to the C-terminal region of the DNA binding and competence protein ComEC as well as ComEA of *B*. *subtilis* (with *E* values of 5e-29 and 2e-06, respectively). In the case of the four other putative proteins, three are homologs of proteins in *B*. *subtilis*: Lmo0944 exhibits similarity to the YneR protein (*E* value 6e-18), Lmo1622 shares homology with the YXKO protein (*E* value 4e-21), and Lmo1065 is homologous to protein YktB (*E* value 2e-37). The other protein, Lmo1211 is highly similar to hypothetical bacterial proteins of unknown function.

**Table 3:** **Penicillin G-inducible genes of *L*. *monocytogenes *identified using the pAT28-*hly *promoter-trap system**

**Strain**	**Gene**	**Comments on encoded protein**^***a***^	**Function of encoded protein**^***b***^
15	*lmo1941*	Contains a LysM domain	Unknown
18	*lmo2820 (axyR)*	Contains a conserved helix-turn-helix DNA-binding domain (HTH_AraC) and a β-D-xylosidase domain (XynB)	Putative transcriptional regulator
37	*lmo1660* (*leuS*)	Contains two catalytic core domains of leucyl tRNA synthetase (LeuRS_core) and an anticodon-binding domain	Leucyl-tRNA synthetase
41	*lmo0943* (*fri*)	Contains a DNA protecting under starvation domain (DPS)	Non-heme iron-binding ferritin
	*lmo0944*	Contains a domain found in a family of proteins involved in iron-sulfur cluster biosynthesis (Fe-S_biosyn)	Unknown
	*lmo0945*	Contains a metallo-beta-lactamase domain (Lactamase_B)	Unknown
198	*lmo1622*	Contains a YXKO-related domain, belongs to the ribokinase-like superfamily	Unknown
199	*lmo2501* (*phoP*)	Contains a CheY-like receiver domain and a winged-helix DNA-binding domain	Two-component response phosphate regulator
201	*lmo1211*	Contains a bacterial domain of unknown function (DUF606)	Unknown
203	*lmo1065*	Contains a bacterial domain of unknown function (DUF1054)	Unknown

^*a*^ Based on data available from the NCBI (http://www.ncbi.nlm.nih.gov/).

^*b*^ Functions are based on annotations provided by the ListiList (http://genolist.pasteur.fr/ListiList/).

### Transcriptional analysis of the identified genes in the presence of penicillin G

**Figure 2:**
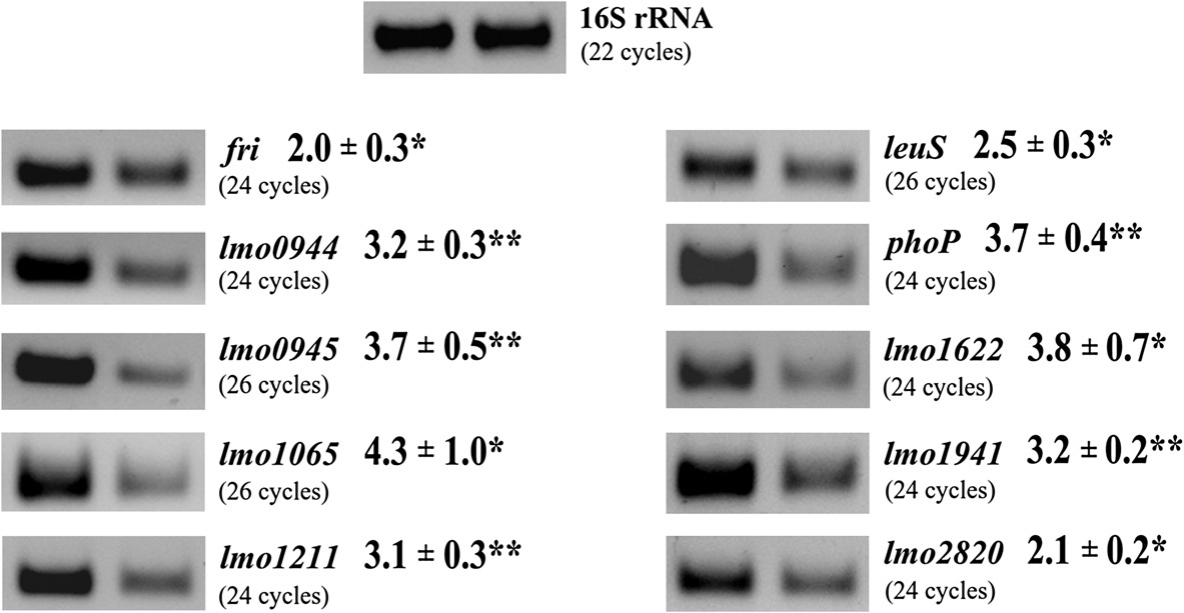
**Transcriptional analysis of gene expression under penicillin G pressure using RT-PCR.** Total RNA was isolated from exponential-phase cultures of *L*. *monocytogenes* EGD grown in BHI broth at 37°C without antibiotics (right) or in the presence of penicillin G at a concentration of 0.09 μg/ml for 30 min (left). The RNA was used as the template in RT reactions with p(dN)6 random primers and the obtained cDNAs were then used in PCRs with a panel of gene-specific primer pairs. All PCRs were performed three times using cDNAs transcribed from three separate RNA preparations, with similar results. In all cases, control PCRs were performed to confirm the complete removal of DNA from the RNA preparations prior to reverse transcription (data not shown). The RT-PCR products were quantified by measuring the level of band fluorescence using ImageQuant software and these values were normalized to those of a 16S rRNA gene fragment amplified in control reactions. The numbers given are the relative amounts of the RT-PCR products obtained for the studied genes using a template of total RNA isolated from wild-type *L*. *monocytogenes* EGD grown in the presence of penicillin G in comparison with the corresponding amounts for this strain grown without antibiotics. Asterisks indicate significant differences according to Student’s *t* test (^*^, *P* < 0.05; ^**^, *P* < 0.01).

To verify penicillin G-inducible expression of the identified genes in wild-type *L*. *monocytogenes* EGD, transcriptional analysis in non-stressed cells and in cells growing under penicillin G pressure was performed, and their relative expression levels were quantified (Figure 2). This analysis confirmed that the annotated genes downstream of the captured DNA in each clone were indeed upregulated in response to the presence of penicillin G, thus validating the results obtained with the *hly* reporter system. In addition, the transcriptional analysis also demonstrated that the genes identified on the basis of elevated reporter gene expression in the presence of penicillin G during the stationary phase of growth, were also induced by this antibiotic in the exponential phase of growth.

### Antimicrobial susceptibility of *L*. *monocytogenes* Δ*fri*, Δ*phoP* and Δ*axyR* mutant strains

**Figure 3:**
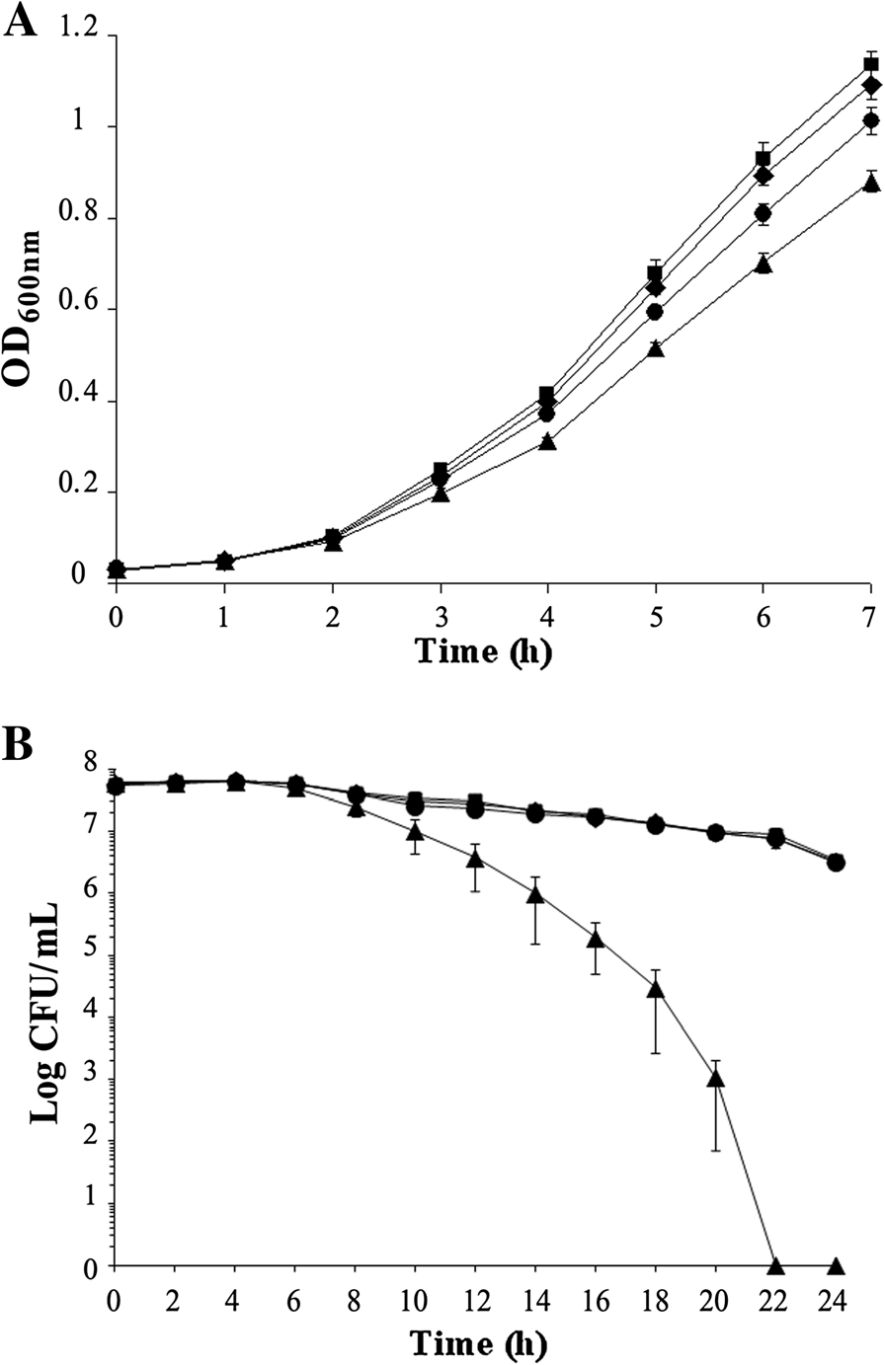
**Growth and survival of *L*. *monocytogenes *strains in sublethal and lethal concentrations of penicillin G.** (**A**) Growth of wild-type *L*. *monocytogenes* EGD (*black circle*), the Δ*axyR* mutant (*black diamond*), Δ*phoP* mutant (*black square*) and Δ*fri* mutant (*black triangle*) in sublethal concentration of penicillin G. BHI broth supplemented with penicillin G (0.09 μg/ml) was inoculated with an overnight culture of each strain (1:100) and incubated with shaking at 37°C. Cell growth was measured spectrophotometrically by determining the OD_600_. (**B**) Survival of wild-type *L*. *monocytogenes* EGD (*black circle*), the Δ*axyR* mutant (*black diamond*), Δ*phoP* mutant (*black square*) and Δ*fri* mutant (*black triangle*) in a lethal concentration of penicillin G. BHI broth supplemented with 32 μg/ml penicillin G was inoculated with a mid-exponential culture of each strain (5 × 10^7^ CFU/ml) and incubated with shaking at 37°C. Viable cell counts were performed by plating serial dilutions of culture samples onto BHI agar and counting colonies after 24–48 h incubation at 37°C. The mean values from three independent experiments are plotted and the error bars represent the standard deviation.

To investigate whether any of the identified genes play a role in the susceptibility of *L*. *monocytogenes* to β-lactams, three of them, namely *fri*, *phoP* and *axyR*, were selected for further study. The Δ*fri* mutant was constructed in a previous study [18], while the Δ*phoP* and Δ*axyR* mutants were created using the temperature-sensitive shuttle vector pMAD via double-crossover homologous recombination. Prior to detailed investigations, the growth rates of the mutants and the parent strain in BHI broth at 37°C were compared, but no differences were observed (data not shown). To determine whether disruption of the *phoP*, *axyR* and *fri* genes affected the susceptibility of *L*. *monocytogenes* to penicillin G and ampicillin – the antibiotics of choice for the treatment of listerial infections [2] – MIC values were determined for the mutants, as was their ability to grow and survive in the presence of sublethal and lethal concentrations of these β-lactams, respectively. The absence of *phoP*, *axyR* or *fri* expression had no effect on the MICs of penicillin G and ampicillin, which were identical for all strains (0.125 μg/ml and 0.25 μg/ml, respectively). However, when the ability of the mutants to grow in a sublethal concentration of penicillin G was examined, the Δ*phoP* and Δ*axyR* mutants were found to grow slightly faster than the wild type, whereas the growth of Δ*fri* was impaired (Figure 3A). The same pattern of growth was observed with a sublethal concentration of ampicillin (data not shown). The tolerance assay revealed that the ability of the Δ*phoP* and Δ*axyR* mutants to survive in the presence of a lethal concentration of penicillin G was no different to that of the wild type, whereas the tolerance of Δ*fri* was significantly impaired since viable cells of this mutant strain could not be recovered after prolonged exposure to penicillin G (Figure 3B). The same pattern of tolerance of the strains to ampicillin was observed (data not shown). To determine whether *phoP*, *axyR* or *fri* play a role in the susceptibility to *L*. *monocytogenes* to β-lactams other than penicillin G and ampicillin, the wild-type strain and the three mutants were tested in an antibiotic disk assay with cephalosporin, monobactam and carbapenem disks. This assay did not reveal any significant alterations in the resistance of *L*. *monocytogenes* to these antibiotics caused by the lack of functional *phoP* or *axyR* genes, but significantly greater zones of growth inhibition were observed for the *fri* mutant with the antibiotics cefalotin and cephradine (data not shown). The MICs of these specific cephalosporin antibiotics were then determined for *L*. *monocytogenes* EGD and the Δ*fri* mutant. In confirmation of the antibiotic disk assay result, the MIC of cefalotin for EGD and Δ*fri* was 2 μg/ml and 1 μg/ml, respectively, whereas the MIC of cephradine for EGD and Δ*fri* was 64 μg/ml and 32 μg/ml, respectively. Thus, interruption of the *fri* gene caused a 2-fold increase in the sensitivity of *L*. *monocytogenes* to these cephalosporins.

## Discussion

In this study, we attempted to identify penicillin G-inducible genes of *L*. *monocytogenes*, some of which might be essential for the survival and growth of this bacterium in the presence of cell wall-acting antibiotics. A promoter trap system was used to identify nine strains showing significantly increased expression of a reporter gene (*hly*) in the presence of penicillin G. In one case (strain 195), the predicted polarity of transcription was opposite to that of *hly*. The cloned sequence corresponded to fragments of the genes *lmo2095* and *lmo2096*, both of which are involved in the metabolism of carbohydrates. A recent study examining the transcription of the entire genome of *L*. *monocytogenes* has shown that the identified promoter drives the transcription of a long antisense RNA with no known physiological role [19].

Analysis of the chromosomal DNA fragments trapped in the other strains permitted the identification of ten penicillin G-inducible genes. Increased expression of the identified genes in the presence of penicillin G was further confirmed by transcriptional analysis. The transcription of seven of the identified genes, namely *lmo1065*, *lmo1211*, *lmo1622*, *leuS*, *lmo1941*, *phoP* and *axyR*, appeared to be upregulated in response to this stress in a growth phase-independent manner, since they were initially identified in the stationary phase of growth and subsequently their elevated expression was also observed in exponentially growing cells. On the basis of the initial promoter trap system results it was difficult to determine whether the genes *fri*, *lmo0944* and *lmo0945*, or only one or two of them, show increased expression under penicillin G pressure in the stationary phase of growth. However all three of these genes were definitely transcriptionally upregulated in response to this stress in the exponential phase of growth.

The functions of the proteins encoded by six of the identified genes are unknown, but four have established functions. One of them, *fri*, encodes a ferritin-like protein which belongs to the Dps family. Previously, this listerial ferritin was shown to contribute to virulence and to play a role in protection against multiple stresses [18,20]. The expression of the *fri* gene is known to be upregulated in a σ^B^-dependent manner [21]. Interestingly, SigB was found to determine the tolerance of *L*. *monocytogenes* to cell envelope-acting antimicrobial agents [12], and in a Δ*fri* mutant strain, overexpression of an anti-sigma B factor, RsbW, was observed [20], which strongly suggests possible modulation of SigB activity by ferritin. Gene *phoP*, a member of the phosphate starvation two-component regulatory system PhoP-PhoR is involved in the regulation of alkaline phosphatase genes in response to environmental signals. In *B*. *subtilis*, it has been shown that the PhoP-PhoR system is also involved in controlling the biosynthesis of teichoic acid, a key component of the cell walls of gram-positive bacteria [16]. More recently, it was found that a lack of *phoR* in *L*. *monocytogenes* results in altered tolerance to ethanol stress. This observation suggests that the listerial PhoP-PhoR system is involved in regulating the composition of the cell wall [22]. Gene *axyR* encodes a putative bimodular protein with an N-terminal region containing a conserved HTH domain required for transcriptional regulation by AraC/XylS regulators at targeted promoters [17]. Some members of the AraC/XylS family are involved in tolerance to antibiotics and others regulate virulence genes. It has been hypothesized that AxyR regulates the expression of the *L*. *monocytogenes* virulence factor InlJ during *in vivo* infection [23], and the contribution of this protein to virulence is in line with the observed upregulation of *axyR* expression during *in vitro* infection [24]. Taking into account the strong indications of their potential role in the response of *L*. *monocytogenes* to β-lactam pressure, these three genes were selected for further study.

Analysis of Δ*axyR* and Δ*phoP* mutant strains revealed that the absence of these gene products had no effect on the MIC values and ability of *L*. *monocytogenes* to survive in the presence of a lethal dose of β-lactams, indicating that these proteins do not play a significant role in the susceptibility and tolerance of this bacterium to these antibiotics. The only difference between these mutant strains and the wild-type was their slightly faster growth in the presence of sublethal concentrations of penicillin G and ampicillin. Under these conditions, cells normally sense damage to the cell wall and respond by significantly reducing their growth rate. We assume, therefore, that the regulators PhoP and AxyR are involved in transmitting signals to adjust the rate of growth under these adverse conditions.

The experiments examining the role of listerial ferritin in the sensitivity and tolerance of *L*. *monocytogenes* to β-lactams produced interesting results. The tolerance of the Δ*fri* mutant to penicillin G and ampicillin was found to be dramatically lower than that of the wild-type strain. The recent study of Kohanski et al. [25] indicated that there is a strong correlation between the ability of bacteria to survive antibiotic action and the level of hydroxyl radicals in antibiotic-treated cells. Efficient killing of bacteria was observed for those antibiotics that cause increased cellular production of H_2_O_2_, which is the end product of an oxidative damage cellular death pathway involving stimulation of the Fenton reaction [25]. On the other hand, Dps proteins are iron-binding and storage proteins that protect cells from oxidative damage by removing excess ferrous ions from the cytosol, making them unavailable for participation in the Fenton reaction [26]. Therefore, it is likely that the impaired β-lactam tolerance of *L*. *monocytogenes* lacking the Dps protein Fri results from its inability to prevent the cellular production of hydroxyl radicals. This hypothesis is supported by a recent study which showed that a Dps protein protects *Salmonella enterica* from the Fenton-mediated killing mechanism of bactericidal antibiotics [27]. It is noteworthy that the Δ*fri* mutant strain also exhibited increased sensitivity to some cephalosporins – antibiotics to which *L*. *monocytogenes* shows high innate resistance – that are often used as the first choice when treating infections of unknown etiology.

Interestingly, transcriptional analysis demonstrated that two of the identified penicillin G-inducible genes, *lmo0944* and *lmo0945*, are cotranscribed with *fri*. This is the first evidence that a gene encoding a Dps protein is transcribed together with downstream genes. As mentioned above, the role of Fri in the stress response and virulence is well established, but the functions of Lmo0944 and Lmo0945 and their potential roles in these processes in *L*. *monocytogenes* are currently unknown and will be the subject of future studies. Similarly, further research effort is required in order to clarify the potential role of the other identified penicillin G-inducible genes in tolerance and/or susceptibility of *L*. *monocytogenes* to β-lactam antibiotics.

## Conclusions

Disease outbreaks caused by *L*. *monocytogenes*-contaminated foods and the serious illnesses and fatalities that occur in susceptible individuals highlight the importance of understanding the mechanisms that enable this bacterium to survive antibiotic therapy. The present study resulted in the identification of ten penicillin G-inducible genes of *L*. *monocytogenes*. In-depth examination of the contribution of three of the identified genes, namely *fri*, *phoP* and *axyR*, to the susceptibility and tolerance of *L*. *monocytogenes* to β-lactams indicated that the regulators PhoP and AxyR do not play a significant role in these reactions. However, these proteins are probably involved in transmitting signals to adjust the rate of growth of *L*. *monocytogenes* under β-lactam pressure. The most important finding of this research is that the ferritin-like protein Fri contributes to *L*. *monocytogenes* tolerance of the β-lactam antibiotics penicillin G and ampicillin – the current drugs of choice for the treatment of listeriosis – as well as to the high innate resistance of this bacterium to some cephalosporins. It is therefore possible that the functions of Fri are essential for the survival of *L*. *monocytogenes* in the clinical setting. In light of the key role of *L*. *monocytogenes* Fri, both in the response to multiple stresses and during infection in vivo, it may represent an attractive target for the development of improved control and treatment strategies for this important pathogen.

## Methods

### Bacterial strains, media, plasmids and DNA techniques

*Escherichia coli* strain DH5α used in cloning experiments was grown on Luria-Bertani medium. The *L*. *monocytogenes* EGD (serotype 1/2a) wild-type strain was kindly provided by S.J. Foster, University of Sheffield, United Kingdom. Isogenic EGDΔ*hly*, EGDΔ*phoP* and EGDΔ*axyR* deletion mutants were constructed in this study (described in detail below), while the isogenic EGDΔ*fri* deletion mutant was a generous gift from Hanne Ingmer, Royal Veterinary and Agricultural University, Denmark. *L*. *monocytogenes* strains were grown in brain heart infusion (BHI) broth medium (Oxoid). *L*. *monocytogenes* EGD and EGDΔ*hly* strains were transformed with different recombinant plasmids by electroporation as described previously [28]. Antibiotics were added to growth media at the following concentrations: ampicillin, 100 μg/ml; chloramphenicol, 10 μg/ml; erythromycin, 5 μg/ml; penicillin G, 0.03 or 0.09 μg/ml; and spectinomycin (SPC), 60 μg/ml. The isolation of chromosomal and plasmid DNA, restriction enzyme analysis, and PCR were performed according to standard protocols [29]. PCR and RT-PCR primers used in this study are listed in Table 4.

**Table 4:** Primers used in this study

**Primer**	**Sequence [5**^**′**^** → 3**^**′**^**]**
16S RNA-E^*a*^	TTAGCTAGTTGGTAGGGT
16S RNA-B^*a*^	AATCCGGACAACGCTTGC
0943F^*ab*^	CATTGGTATATGAGAGGCCAC
0943R^*ab*^	CATTGTCGCCTTCTTTGTCAG
0944F^*ab*^	ATGGTTTCATGATGAGTTTGATGT
0944R2^*b*^	ATTTTCCAGTCGTGGTCTTTG
0944R^*ac*^	TCCGTTTTTGGTTCATAGTCG
0945F^*ab*^	CCGCACGCAGACCATATTG
0945R2^*b*^	ATTGGCACCGCTATCTACC
0945R^*ac*^	CTGGTTGGATGTGGACGATC
1065F^*a*^	GCTTGAAGCACGCATGACC
1065R^*a*^	GCCGTCATGCACAGGATAC
1211F^*a*^	CAGGTTTGTTAGCTGGGATG
1211R^*a*^	ACGCCAAGTAGACGTTCGA
1622F^*a*^	TAGCGTCAACCGTCCTGCT
1622R^*a*^	ATCTCCCATACCGCCAGTG
1660F^*a*^	TACCGCGTACGCAGATCG
1660R^*a*^	GAATCAACACGTAGTCCGC
1941F^*a*^	CCGGCTGATTATGACATGAG
1941R^*a*^	TGCTTTCTCGGCAGCAGC
2501F^*a*^	GTGGTGACAGCTGAAGATG
2501R^*a*^	GTGGTGACAGCTGAAGATG
2820F^*a*^	GCCTTGTCGCTTCGTGTG
2820R^*a*^	ACTAAGACAACGGGCAGTC
llo-1^*d*^	CG**GGTACC**AGGTAGAGCGGACATCCATTG
llo-2^*e*^	GTTTTAGGATCCCCCGGGGGGTTTCACTCTCCTTCTAC
llo-3	CCCGGGGGATCCTAAAACCGCTTAACACACACG
llo-4^*f*^	GCG**TCTAGA**TTCTTCCCCGACAGAATCTGC
phoP-1^*g*^	CA**GGATCC**AGTTTTGGGTGCTCGTGC
phoP-2^*h*^	TC**GAATTC**CTATCTACCATCTTCAGCTGTCAC
phoP-3^*h*^	TC**GAATTC**GGACTTGAACTTGGAGCAG
phoP-4 ^*i*^	CG**TCCATGG**TTACGTTCTCCATTTTATAACCG
axyR-1 ^*g*^	CA**GGATCC**GGTAGCGATTAATTTTCACGAC
axyR-2 ^*h*^	TC**GAATTC**CTAATCATTGACTTCTTTCCTAGCAGA
axyR-3 ^*h*^	TC**GAATTC**CTTATGCTAGTGAACTGGAATAC
axyR-4 ^*i*^	CTC**CCATGG**CCGTAATCGTCTCATCGCTC
Hly-1 ^*g*^	GCG**GGATCC**TGTAGAAGGAGAGTGAAACCCATG
Hly-2 ^*j*^	GCG**GTCGAC**ACAATTATTCGATTGGATTATCTAC
seq-1	CAGGAAACAGCTATGACCATG
seq-2	ACTAATATAAGTGTAATAAAAACTAGCAT

^*a*^ Primers used for analysis of gene expression under stress conditions.

^*b*^ Primers used for PCR in cotranscription analysis.

^*c*^ Primer used for reverse transcription in cotranscription analysis.

^*d*^ The sequence in boldface type is the KpnI restriction enzyme site.

^*e*^ The underlined sequence is an overhang complementary to primer llo-3.

^*f*^ The sequence in boldface type is the XbaI restriction enzyme site.

^*g*^ The sequence in boldface type is the BamHI restriction enzyme site.

^*h*^ The sequence in boldface type is the EcoRI restriction enzyme site.

^*i*^ The sequence in boldface type is the NcoI restriction enzyme site.

^*j*^ The sequence in boldface type is the SalI restriction enzyme site.

### Construction and analysis of *L*. *monocytogenes* genomic libraries

Two ~400-bp DNA fragments flanking the *L*. *monocytogenes hly* gene were amplified by PCR using strain EGD chromosomal DNA as the template. The primers used to amplify the *hly* 5^′^ flanking fragment were llo-1 and llo-2, and those for the 3^′^ fragment were llo-3 and llo-4. The amplified 5^′^ and 3^′^ fragments were ligated by PCR using primers llo-1 and llo-4, and following digestion with KpnI and XbaI, the product was cloned into the corresponding restriction sites of the thermosensitive plasmid pKSV7 [30], yielding pKSV7Δ*hly*. This plasmid was introduced into *L*. *monocytogenes* EGD by electroporation and gene replacement was performed as described previously [30]. Chloramphenicol-sensitive clones were screened for the presence of the *hly* deletion by PCR with primers llo-1 and llo-4. A shorter PCR product was amplified from strains that had undergone allelic exchange to introduce the deleted version of the wild-type allele on the chromosome. The *hly* deletion was further verified by DNA sequencing and the absence of a hemolytic phenotype during growth of bacteria on BHI agar medium supplemented with 5% sheep blood.

The *hly* gene preceded by its ribosome binding site was amplified by PCR from strain EGD chromosomal DNA using the primer pair Hly-1 and Hly-2. DNA Polymerase pfu (Fermentas) was used in the PCR. The amplified fragment was digested with BamHI and SalI and cloned using the corresponding restriction sites into the high-copy-number *E*. *coli*-gram positive bacteria shuttle vector pAT28 [31] to produce plasmid pAT28-*hly*. The *hly* sequence cloned in pAT28-*hly*, used for the generation of libraries, was confirmed by DNA sequencing.

Four genomic DNA libraries were constructed in pAT28-*hly*. Chromosomal DNA from *L*. *monocytogenes* EGD was mechanically sheared using a nebulizer according to the manufacturer’s instructions (Invitrogen) or was partially digested with restriction endonucleases BsuRI, Bsh1236I or simultaneously with BsuRI and Bsh1236I. In each case, the fragmented DNA was separated by gel electrophoresis and fragments with a size distribution from 500 to 2000 bp were excised from the gel and purified. In the case of the DNA fragments obtained by nebulization, the ends were blunted by treatment with T4 DNA polymerase (Fermentas). All four DNA fragment pools were then cloned into the SmaI site of pAT28-*hly* using a two-step ligation procedure [32]. After purification, each plasmid library was introduced into *L*. *monocytogenes* strain EGDΔ*hly* by electroporation. The transformants were plated on BHI-SPC agar supplemented with 5% defibrinated sheep blood and penicillin G (0.03 μg/ml), and incubated overnight at 37°C. Approximately 2.3 × 10^3^, 1 × 10^4^, 3 × 10^3^ and 6.7 × 10^3^ recombinant *L*. *monocytogenes* were obtained for the libraries created using DNA fragmented by nebulization, BsuRI, Bsh1236I or simultaneous BsuRI and Bsh1236I digestion, respectively. Among these clones, the frequencies of hemolytic colonies were 0.6%, 1.1%, 2.6% and 0.9%, respectively. The total number of hemolytic clones identified was 259.

All hemolytic clones were replica plated on BHI-SPC agar supplemented with 5% defibrinated sheep blood alone, and on BHI-SPC agar supplemented with 5% defibrinated sheep blood plus penicillin G (0.03 μg/ml). After overnight incubation at 37°C, the diameter of zones of hemolysis created by each clone during growth on plates with and without penicillin G was compared. The clones that showed increased hemolysis in the presence of this antibiotic were subjected to further analysis.

### Hemolytic activity assay

*L*. *monocytogenes* strains were grown in BHI-SPC medium overnight with shaking at 37°C. The following morning, each culture was diluted 1:20 into fresh medium in duplicate. These cultures were grown at 37°C with aeration to an optical density at 600 nm (OD_600_) of 0.5. At this point, penicillin G was added to a final concentration of 0.03 μg/ml to one of the duplicate cultures and the incubation was continued for a further 2 hours, when the cells reached early stationary phase. The number of viable bacteria present in both cultures was determined by plating serial dilutions onto BHI-SPC agar and counting the colonies after overnight incubation at 37°C. The hemolytic activity in the supernatants from both cultures was assayed by determining the level of hemoglobin released from sheep red blood cells (SRBC), essentially as described previously [33]. Briefly, a 1 ml sample of culture was centrifuged to pellet the cells and 20 μl of the supernatant was added to 1 ml of PBS (phosphate-buffered saline, pH 5.6) containing SRBC, and this was incubated at 37°C for 30 min. To avoid complete hemolysis, the final amount of SRBC used in the assay was 0.5%, 1% or 2%, and was individually determined for each strain. The reactions were then centrifuged to pellet unlysed cells and the hemoglobin absorbance in the supernatants was measured at 410 nm. Hemolytic activity was expressed as the percentage of complete hemolysis, which was determined by lysing appropriate amounts of SRBC with 1% Triton X-100 per 10^9^ bacteria. The presented results are the average of at least three independent experiments, each carried out in triplicate.

### Sequence analysis

Chromosomal DNA fragments inserted upstream of *hly* in pAT28-*hly* derived plasmids were sequenced with the primers seq-1and seq-2. The sequences were compared with the *L*. *monocytogenes* EGD-e genome using the BLAST program on the NCBI website.

### Total RNA isolation

For RNA isolation, a culture was inoculated with a single colony of wild-type *L*. *monocytogenes* EGD and incubated overnight at 37°C. The following morning, the culture was diluted 1:50 into fresh medium in duplicate. These cultures were grown at 37°C with aeration to an OD600 of 0.4. At this point, penicillin G was added to a final concentration of 0.09 μg/ml to one of the duplicate cultures and incubation at 37°C was continued for an additional 30 min. Total RNA was isolated using the hot acid phenol procedure [34]. Briefly, 1 ml of the separate cultures was centrifuged (12,000 × g for 30 s) and the cell pellets were immediately resuspended in ice-cold lysis buffer (20 mM sodium acetate, 1 mM EDTA, 1% sodium dodecyl sulfate, pH 5.2). Each cell lysate was added to an equal volume of preheated (65°C) acid phenol-chloroform-isoamyl alcohol with 200 mg of glass beads and placed in a heating block (65°C) for 10 min with frequent vortexing. The suspensions were centrifuged and the aqueous phase was extracted twice with hot acid phenol-chloroform-isoamyl alcohol, followed by single extraction with 100% chloroform and precipitated in ice-cold ethanol for at least 1 h. Nucleic acid precipitates were pelleted by centrifugation (14,000 × g for 15 min), washed with 70% ethanol and resuspended in diethyl pyrocarbonate (DEPC)-treated water. Contaminating DNA was degraded using RNase-free DNase (Fermentas) following the manufacturer‘s instructions, except that incubation at 37°C was prolonged to 2 h. The concentration and purity of the RNA preparations was then estimated by measuring the A_260_ and A_280_ with a NanoDrop ND-1000 spectrophotometer. The RNA quality and integrity was further analyzed by agarose gel electrophoresis. The absence of DNA from RNA preparations was verified by the failure to amplify a 16S rRNA gene fragment in a 30-cycle PCR using 1 μg of RNA as the template. The prepared RNA was stored at −70°C until required for analysis.

### Transcriptional analysis of the identified genes

To compare the level of transcription of the identified genes in non-stressed cells and in cells growing under penicillin G pressure, reverse transcriptase-PCR (RT-PCR) was performed, essentially as described previously [35]. Briefly, 100 ng of total RNA were converted to cDNA using RevertAid H Minus M-MuLV reverse transcriptase (Fermentas) and p(dN)6 random primers following the manufacturer‘s instructions. PCRs were performed using one-twentieth of the obtained cDNAs as the template with primers specific for the identified genes and for the 16S rRNA gene (listed in Table 4). To permit optimal quantification of PCR products, the reactions were subjected to 16, 22 or 30 thermal cycles before the amplified bands were visualized by agarose gel electrophoresis. The RT-PCR products were quantified by densitometric analysis of DNA bands on gel images using ImageQuant™ TL software (GE Healthcare, United Kingdom). For cotranscription analysis of the *fri*, *lmo0944* and *lmo0945* genes, reverse transcription was performed using primer 0945R specific for the *lmo0945* gene and primer 0944R specific for the *lmo0944* gene. The obtained cDNAs were then used as the template for PCR performed with primers specific for internal fragments of the *fri*, *lmo0944* and *lmo0945* genes. The expected sizes of the products were 288 bp, 212 bp and 332 bp for *fri*, *lmo0944* and *lmo0945*, respectively.

### Construction of *L*. *monocytogenes* strains with *phoP* and *axyR* deletions

For the construction of in-frame mutants with deletions of *phoP* and *axyR*, *L*. *monocytogenes* EGD chromosomal DNA was used as the template for the PCR amplification of DNA fragments representing either the 5^′^ end and upstream sequences or the 3^′^ end and downstream sequences of the respective genes. Primer pair phoP-1 and phoP-2 was used for amplification of a ~500 bp 5^′^ fragment, and primer pair phoP-3 and phoP-4 was used for amplification of a ~450 bp 3^′^ fragment of the *phoP* gene. For the *axyR* gene, primer pair axyR-1 and axyR-2, and primer pair axyR-3 and axyR-4 were used for amplification of ~550 bp 5^′^ and ~580 bp 3^′^ fragments, respectively. The amplified 5^′^ fragments of *phoP* and *axyR* were digested with BamHI and EcoRI and cloned into the thermosensitive plasmid pMAD using the corresponding restriction sites. Using the EcoRI and NcoI sites in the plasmids and fragments, the resulting constructs were used to clone the amplified 3^′^ fragments of *phoP* and *axyR* downstream of the 5^′^ fragments of the appropriate genes, yielding constructs pMADΔ*phoP* and pMADΔ*axyR*, respectively. These plasmids were introduced into *L*. *monocytogenes* EGD by electroporation and gene replacement was performed as described previously [36]. Erythromycin-sensitive clones were screened for the presence of the *phoP* and *axyR* deletion by PCR with primers phoP-1 and phoP-4, and primers axyR-1 and axyR-4, respectively. The shorter PCR products amplified from these strains were sequenced to verify that they carried the desired deletions.

### Antibiotic susceptibility tests

The susceptibility of *L*. *monocytogenes* strains to different antibiotics was examined using a microdilution test. The antimicrobial agents were obtained as powders (Sigma-Aldrich, St. Louis, USA) and stock solutions were prepared immediately before use. The microdilution method was performed according to guidelines of the Clinical and Laboratory Standards Institute [37]. Briefly, an overnight culture of each strain was serially diluted in BHI broth to a cell density of 10^5^ CFU/ml and 100 μl aliquots were added to the wells of 96-well microdilution plates containing 100 μl of two-fold dilutions of the different antimicrobial agents in BHI broth. These plates were then incubated at 37°C for 18–22 h before the MIC endpoints were read. The MIC was determined as the lowest antibiotic concentration that resulted in the absence of apparent growth of the bacteria. MIC determinations were carried out in triplicate. For quality control of performance and reliability of the results of MIC determination, standard *Escherichia coli* ATCC 25922 and *Staphylococcus aureus* ATCC 25923 strains were used in parallel tests.

The growth of *L*. *monocytogenes* strains in the presence of a sublethal level of penicillin G was examined by plotting growth curves. Overnight cultures were diluted (1:100) into BHI broth supplemented with 0.09 μg/ml penicillin G and incubated with shaking at 37°C. Cell growth was monitored spectrophotometrically by measuring the OD_600_. The presented results are the average of three independent experiments, each carried out in triplicate.

The tolerance of *L*. *monocytogenes* strains to penicillin G was tested as described previously [12], except that cultures in the exponential rather than the stationary phase of growth were used for the assays. Briefly, cultures in mid-exponential phase (OD_600_ 0.4) were diluted (5 × 10^7^ CFU/ml) into BHI broth supplemented with 32 μg/ml penicillin G and incubated with shaking at 37°C. Serial dilutions of the cultures were plated on BHI agar, incubated at 37°C for 24–48 h and the colonies counted to establish the number of viable cells. The presented results are the average of three independent experiments, each carried out in triplicate.

## Competing interests

The authors declare that they have no competing interests.

## Authors’ contributions

AK-B created *L*. *monocytogenes* strains with *phoP* and *axyR* deletions, performed the susceptibility tests as well as conceived and designed the entire study and prepared the manuscript. JM created the reporter system for the generation of *L*. *monocytogenes* genomic libraries. DD and KW carried out the screening of genomic libraries as well as the hemolytic activity assays. AS performed the transcriptional analysis. All authors read and approved the final version of the manuscript.
